# Modeling patient access to therapeutic oxytocin in Zanzibar, Tanzania

**DOI:** 10.1186/s12913-018-3452-8

**Published:** 2018-08-17

**Authors:** Devika Nadkarni, Sara Gravelyn, Monica Brova, Sarem Rashid, Randy Yee, Donovan Guttieres, Katie Clifford, Darash Desai, Muhammad Zaman

**Affiliations:** Department of Biomedical Engineering, Boston University, 38 Cummington Street, Boston, MA 02215 USA

## Abstract

**Background:**

Our objective is to estimate the effects of therapeutic oxytocin supply chain factors and social determinants of health on patient access to oxytocin in low-income settings using system dynamics modeling. Postpartum hemorrhage (PPH), a major cause of maternal mortality disproportionately affects women in low and middle income countries (LMICs). The World Health Organization recommends therapeutic oxytocin as the frontline uterotonic for PPH management and prevention. However, lack of access to quality therapeutic oxytocin in Tanzania, and throughout Sub-Saharan Africa, continues to result in a high number of preventable maternal deaths.

**Methods:**

We used publicly available data from Zanzibar and Sub-Saharan Africa, literature review, oxytocin degradation kinetics and previously developed systems dynamics models to understand the barriers in patient access to quality therapeutic oxytocin.

**Results:**

The model makes four basic predictions. First, there is a major gap between therapeutic oxytocin procurement and availability. Second, it predicts that at current population increase rates, oxytocin supply will have to be doubled in the next 30 years. Third, supply and storage temperature until 30 °C has minimal effect on oxytocin quality and finally distance of 5 km or less to birthing facility has a small effect on overall access to oxytocin.

**Conclusions:**

The model provides a systems level approach to therapeutic oxytocin access, incorporating supply and procurement, socio-economic factors, as well as storage conditions to understand how women’s access to oxytocin over time can be sustained for better health outcomes.

## Background

Postpartum hemorrhage (PPH), a major cause of maternal mortality, is characterized by excessive bleeding post-delivery and disproportionately affects women in low and middle income countries (LMICs) [1]. Data suggests that nearly 2% of women globally, and up to 10.5% of mothers in Sub-Saharan Africa, experience PPH [2, 3]. In 2014, it was reported there were still 410 maternal deaths per 100,000 live births in Tanzania (compared to 4 maternal deaths per 100,000 in the European Union), with a high number of those deaths attributed to PPH [4, 5].

Lack of access to quality healthcare products and services is a major factor driving maternal mortality in Tanzania. The World Health Organization recommends oxytocin as the frontline uterotonic for PPH management and prevention. However, lack of access to quality therapeutic oxytocin in Tanzania, and throughout Sub-Saharan Africa, continues to result in a high number of preventable maternal deaths [1, 2]. Pregnant women in Tanzania face a variety of challenging factors in accessing quality healthcare, and oxytocin, including infrastructural, socio-cultural, economic and political barriers. Quality healthcare encompasses provision of timely access to effective, safe, equitable, and patient-centered medical care that increases the likelihood of improving patient health. These barriers not only delay a woman’s decision to seek care, but often prevents her from getting quality and timely healthcare when she does seek treatment [1, 6–8].

Inability to access quality healthcare reflects a fractured and inefficient system that contributes to health inequity among the population it serves [6–9]. Poor health outcomes among pregnant women in Tanzania are often exacerbated by substandard infrastructure, lack of medical personnel, inadequate health education, and sociocultural determinants [6–12]. Despite awareness among public health professionals of these multi-sectoral barriers to care, a rigorous study incorporating these factors to analyze patient access to healthcare commodities, such as oxytocin, has yet to be conducted.

System dynamics modeling offers a robust approach to evaluate the effects of technological, infrastructural and socio-economic factors on health systems and has become an increasingly useful tool for understanding the complexities of healthcare delivery in LMICs [6, 13–15]. An effective dynamic model not only provides a realistic representation of a behavior or trend, but allows users to predict future behaviors based on real-world or simulated scenarios [13]. Manipulation of the model illustrates how changes in one or more variables can impact health outcomes, which is often difficult to demonstrate in nonlinear complex systems.

Previous studies have applied system dynamics models in identifying the optimal location of a new health facility by accounting for a host of factors such as population density, existing supply chains, and adequate resources [9, 13, 14]. A number of models have focused on the role of upstream pharmaceutical production and supply chain logistics when examining availability of health commodities [8–10, 13, 14, 16]. However, these models have limited ability to account for downstream supply chain impediments and non-supply chain barriers [9].

Our model aims to bridge this gap by integrating comprehensive supply chain logistics with socioeconomic factors affecting the population to determine the most effective way to optimize therapeutic oxytocin access for women giving birth at Zanzibari health facilities. In this study, oxytocin refers to the medication for prophylactic and therapeutic use, and not the natural peptide oxytocin. By determining the drivers behind improved access to quality oxytocin, both at the facility and national level, this model can provide useful information to policy-makers on how to allocate resources to best improve patient health outcomes. This paper aims to use systems dynamics modeling to gain deeper insight into the availability of and access to oxytocin in Zanzibar, and to predict access trends based on changes in model variables.

## Methods

### Data collection

Zanzibar is an East African archipelago composed of two major islands - Unguja and Pemba. Zanzibar’s health system presents a unique opportunity for development of this model due to its relatively small size, availability of data on the drug delivery system, Geographic Information System (GIS) data, and well-understood gaps in access to quality maternal health care [17–20]. Data for our study was collected from site visits in 2015 and 2016, and documented reports from the Zanzibar Central Medical Store (CMS) regarding oxytocin procurement, and delivery [12, 13, 17, 19, 20]. From this data, temperature logs were incorporated into a decay function to predict natural oxytocin decay. CMS procurement records were used to model the frequency and cost of drug delivery, and to understand stock-outs and patterns in distribution. A systematic literature review was conducted to examine the supply chain structure of similar healthcare systems, and identify factors affecting access to care in sub-Saharan Africa [14, 16, 21–29].

### Model development

**Fig. 1:**
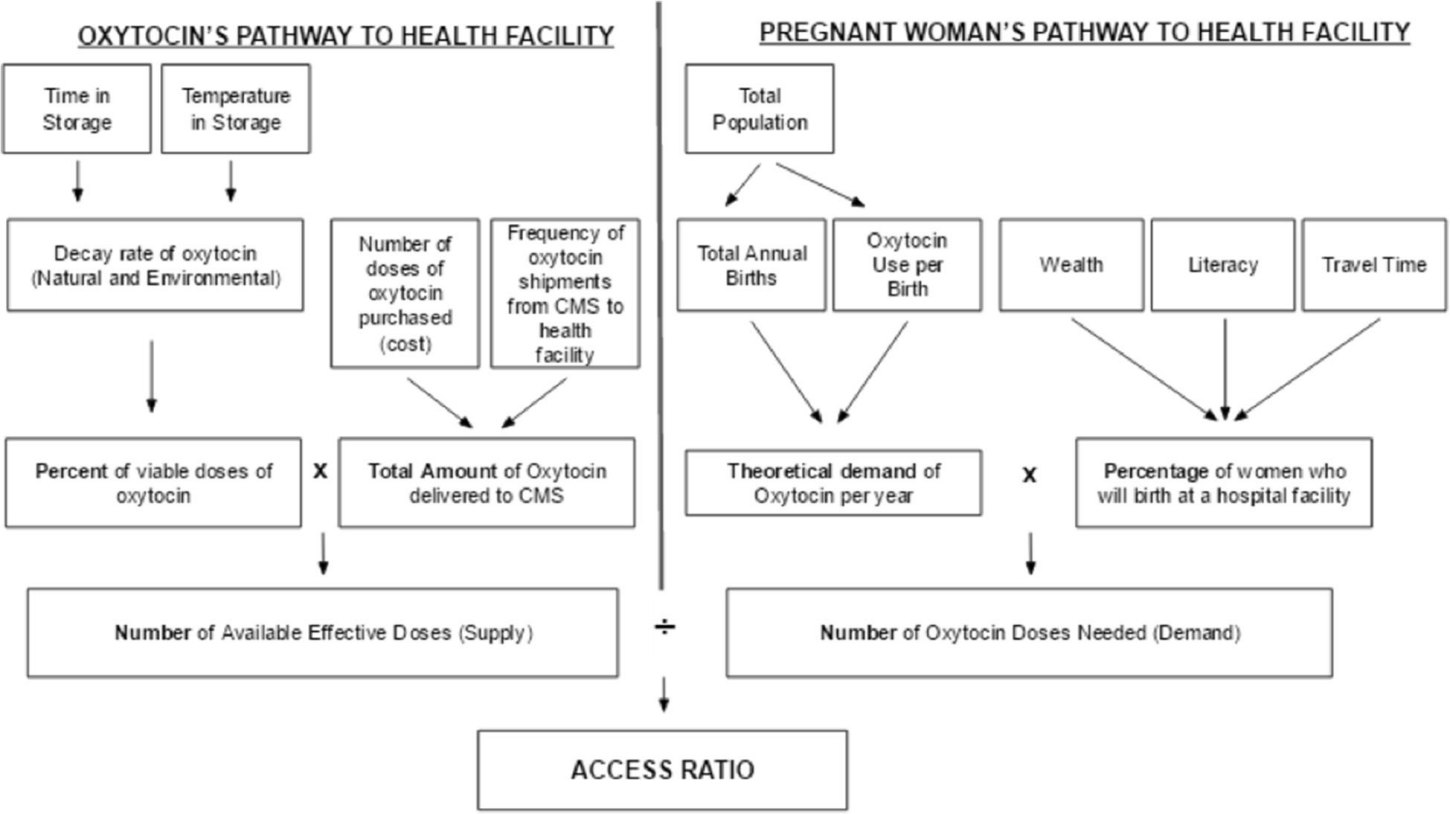
A conceptual framework for this model. Based on the conceptual framework, MATLAB (Mathworks, Inc., Needham, MA) was used to develop a model that tracks the two major aspects involved in patient access to oxytocin - the supply of oxytocin to a health facility in Zanzibar, and the pregnant woman’s ability to reach a health facility in Zanzibar for delivery which generates demand for oxytocin

The collected data was used to develop a conceptual framework (Fig. 1), which was converted into a computational model and analyzed using MATLAB (Mathworks, Inc., Needham, MA). The model incorporates both the supply and demand pathways in oxytocin access: the availability of quality oxytocin, and patient access to it (Fig. 1). Availability describes oxytocin’s journey through the supply chain, from arrival in Zanzibar to distribution to health facilities. Access represents the likelihood a woman will choose to deliver at a health facility. A weekly access ratio is determined. The ‘weekly access ratio’ is defined as the number of oxytocin doses available each week compared to the demand for oxytocin that it must serve for all remaining weeks until the next shipment arrives. User inputs to the model include number of annual births, temperature of oxytocin storage, annual oxytocin supply, and frequency of oxytocin delivery. Annual births and oxytocin supply can be input at a national level, or for a desired catchment area around an Emergency Obstetric and Newborn Care facility (EmONC).

Patient access depends on factors that determine the likelihood a pregnant woman will deliver at birthing facility. Variable selection is based on factors found in the literature that have the most influence on patient access [15].

Tsawe et al. estimate that variables that seem to have a greatest impact on a woman’s access to a birthing facility are distance to the nearest facility, literacy status of the woman, and socioeconomic status of the woman and her family [15]. To quantitatively represent the relationships between these variables, a weighted probability was derived from odds ratios (ORs) of factors influencing maternal health outcomes in Sub-Saharan Africa. The richest wealth quintile was associated with an OR of 2.55, literacy with an OR of 1.99, and rural residence with an OR of 0.82 [15].

The input number of annual births is used to approximate the number of weekly births at the national or facility level. This in turn is used as a measure of the number of women in need of oxytocin each week within a defined region of interest. The model then estimates the proportion of these women that would seek delivery at a health facility. The total national or facility level demand for oxytocin is determined by assuming that each woman receives one dose of prophylactic oxytocin, and that 2% of all women will develop postpartum hemorrhage, requiring four additional doses, as per the standard practice in Zanzibar.

The total weekly demand is used to track oxytocin consumption upon arrival at the health care facility each week. The model assumes there are no delays in arrival of oxytocin shipments to Zanzibar from the manufacturer, nor are there breaks in cold chain storage during shipment to CMS. This assumption is consistent with the records at the Central Medical Stores in Zanzibar, though the limitations of this assumption are discussed in a later section. The model also assumes that demand for oxytocin remains the same each week. It then determines the total number of remaining oxytocin doses each week based on the total supply, frequency of supply, the total weekly demand for oxytocin, and the rate of oxytocin decay.

The model accounts for multiple shipments of a total annual supply of oxytocin. It also accounts for the depreciation of oxytocin quality over the supply chain cycle due to variations in storage temperature, which significantly impacts oxytocin decay rates [30, 31].

1 $$ {k}_{oxytocin}= Ae\frac{- E\alpha}{RT} $$

The decay of a given shipment of oxytocin is tracked weekly and with a rate constant described by a first-order decay for oxytocin at pH 4.5 -- pH at which the drug is most stable (Eq. 1) [27, 28]. The rate constant k_oxytocin_ was calculated from the Arrhenius constant A, the activation energy E_a_ of oxytocin at pH of 4.5, and the ideal gas constant R, each of which was determined by kinetic studies of Hawe et al. [29] The rate constant is multiplied by the concentration of oxytocin in one dose to determine the rate of decay in mg/mL/day. The rate of decay is integrated over a specified interval of time to determine the concentration of oxytocin that has decayed. The decayed concentration is subtracted from the original dose concentration to determine the concentration of viable oxytocin remaining. The viable dose strength is then calculated as percent of viable oxytocin remaining after decay over the given time interval relative to the original concentration of oxytocin in the dose.

To track the quality of available oxytocin, the model inputs temperature and length of time in storage into eq. 1 to determine the amount of viable oxytocin remaining after decay. The viable dose strength for each week is multiplied by the number of total doses in inventory to obtain the number of effective doses. For dose strengths between 50 and 85% of a full strength dose, twice as many doses per patient will be required, halving the number of viable doses remaining. Similarly, for dose strengths between 33 and 54%, three times as many total doses per patient will be required, reducing the number of viable doses by 2/3. A dose strength below 33% assumes zero effective doses meaning the remaining dose stock is not viable.

### Measures of access

The weekly access ratio illustrates the number of effective doses available in a given week compared to the demand for oxytocin available until the next shipment arrives. A weekly access ratio of 1.0 indicates oxytocin supply is exactly meeting weekly demand. The average access ratio is the average of the weekly access ratios over 52 weeks.

The model factors in oxytocin supply shipments, population growth, temperature-dependent oxytocin degradation, and the impact of socioeconomic factors on a woman’s decision to deliver at an EmONC. These variables are used to generate an access ratio of supply (available oxytocin at facility) and demand (women delivering at health facility), allowing the comparison of various scenarios on women’s accessibility to oxytocin at facilities. The variables are shown in Table 1.

**Table 1:** Description of model variables

Variable Name	Type	Description
Inflow of Drugs – Supply Chain from manufacturer to end-user		
Size of Shipment	DV	Weight or freight size of the shipment
Frequency of Shipment	DV	Number of times a shipment occurs within a given period of time
Baseline Cost Prior of Oxytocin Dose	DV	Aggregate cost of standard oxytocin dose
Availability at Facility - Review of depreciation in drug quality		
Quality Depreciation due to Environment (ambient temperature)	DV	Extent of loss in drug quality due to ambient temperature conditions
Natural Rate of Quality Decay	IV	Half-life of drug
Accessibility - Patient Access – describes the patient’s access to health facilities.		
Travel time to health facility	IV	Distance to health center that suits patient’s needs
Literacy	IV	School completion by patient or caregiver
Wealth Quintile	DV	Patient’s access to funds
Patient Access to Health Facility	DV	Fraction of percent of patients able to access adequate health facilities from those who need it.

*Output -* The weekly access ratio is the ratio of the number of effective doses of quality oxytocin available in a given week divided by the demand for oxytocin that must remain available until the next shipment arrives. A weekly access ratio of 1 would indicate oxytocin supply perfectly matched with the current weekly demand

## Results

### Oxytocin supply

**Fig. 2:**
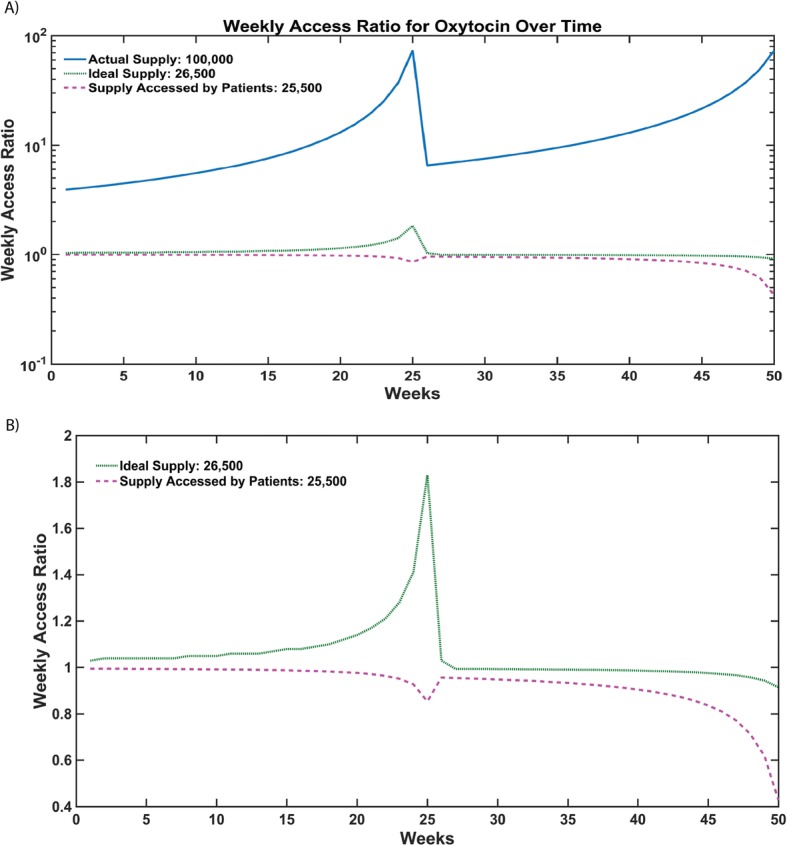
**a** The weekly access ratio for each week during the year, for ideal annual supply values: 100,000 (actual supply arriving at the Central Medical Store, Zanzibar), 26,500 (ideal supply to obtain an average access ratio of 1 ± 0.02, considering 50% of women access the delivery facility), and 25,500 (supply that is predicted to actually reach patients, based on ~ 85% of patients being able to access oxytocin at a facility). (Inset) closer comparison of ideal supply (solid gray) and supply accessed by patients (dotted). **b** Closer comparison of ideal supply and supply accessed by patients

Figure 2 describes the impact of different sizes of annual oxytocin supply to Zanzibar. As per CMS records, 100,000 doses is the actual supply of oxytocin ordered each year. However, the model predicts that 26,500 doses are required for an average access ratio of 1, while 25,500 doses are required for an average access ratio of 0.85. The ratio of 0.85 is used as a benchmark to reflect the larger demand for oxytocin than supply. A peak occurs at Week 25 due to the restocking of the oxytocin inventory at the CMS every 6 months. Just prior to a shipment being received, the amount of oxytocin in stock relative to the number of patients that will need oxytocin before the next shipment is very low -- resulting in a drop in weekly access ratio.

The model estimates the number of doses reaching patients and the number that are unaccounted for can thus be translated to the cost of losing 73.5% of annual national oxytocin supply. Based on the socioeconomic factors previously described, our model predicts that, based on current wealth distribution, literacy rates, and EmONC facility distribution, approximately 50% of Zanzibari women will deliver at a facility. This corroborates with current data that shows the proportion of the female population delivering at facilities is approximately 50% [32]. With 50% of the pregnant population accessing facilities, Zanzibar would require 26,500 oxytocin doses in order to meet the demand and obtain an average access ratio of 1.0. This supply is significantly below the 100,000 doses that is currently ordered by the Ministry of Health through CMS, and still results in shortages of oxytocin at facilities in Zanzibar. Even with 100% of the pregnant population in Zanzibar accessing facilities for delivery, only 53,000 doses would be required to obtain an average access ratio of 1.0. Our model points to bottlenecks and can help to identify inefficiencies in the system that ultimately impede access to oxytocin.

### Oxytocin access and population growth

**Fig. 3:**
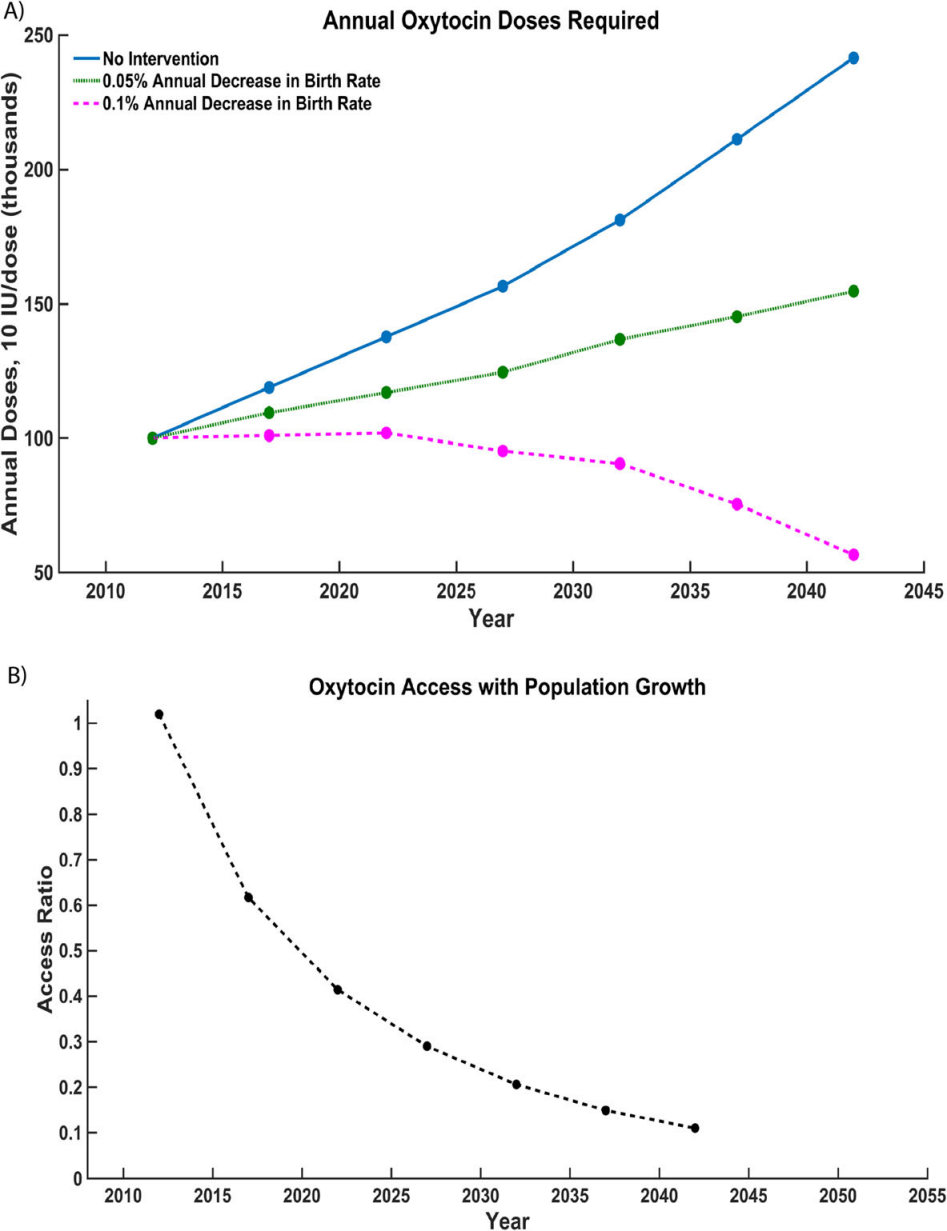
**a** Effect of different rates of population growth (no intervention, 0.05% annual birth decline, 0.1% annual birth decline) on annual oxytocin doses required. **b** Projected decline in oxytocin access ratio assuming a fixed oxytocin supply and a continued annual population growth of 3%

Population growth in Zanzibar occurs at 3% per year, requiring an increasing annual supply of oxytocin to meet patient needs. Our model predicts oxytocin supply would have to double over a period of 30 years in order to obtain an average access ratio of 1 amid current population growth (Fig. 3a). Figure 3b shows the percentage decrease in access ration if the birth rate remains unchanged.

### Oxytocin quality and degradation

**Fig. 4:**
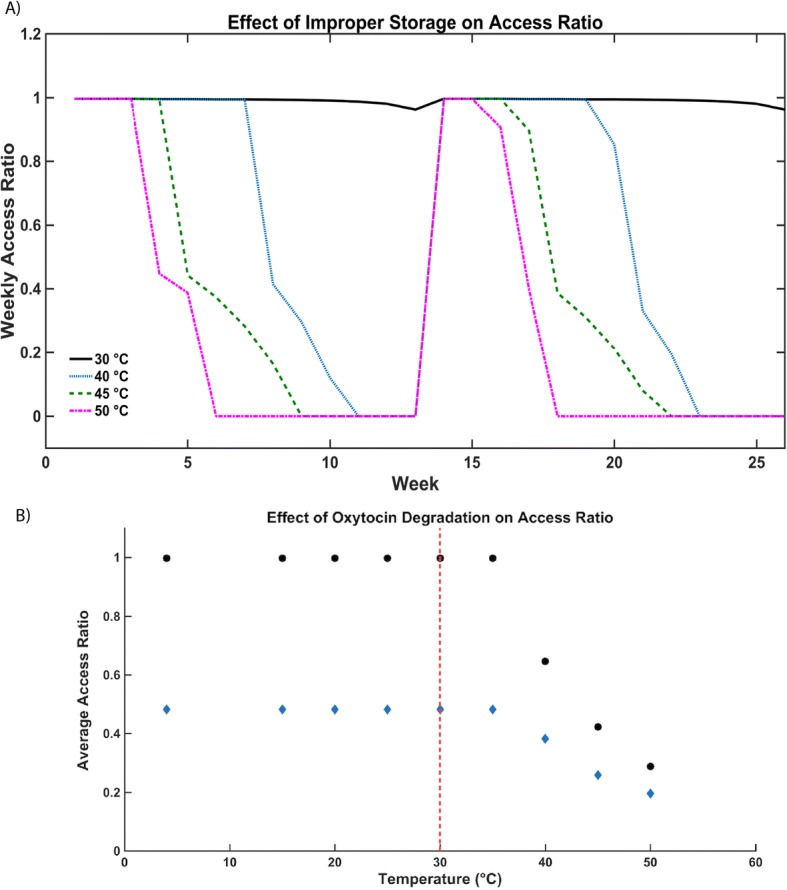
**a** Effect of ambient temperature (up to 50 °C) on oxytocin dose strength and subsequently on average access ratios of 1 (dots) and 0.5 (diamond) respectively. **b** Decline in weekly access ratio over 2 quarters at temperatures of 30 °C (black), 40 °C (green), 45 °C (blue), and 50 °C (pink). Temperatures were based on the ambient temperatures over the year in Zanzibar. Increase in weekly access ratio on week 14 represents arrival of a new shipment of oxytocin at the beginning of a new quarter

While high temperature contributes to quality decay, our model shows that ambient temperature appears to have a negligible effect on oxytocin dose strength until 30 °C, after which it decays rapidly at higher temperatures. Dose strength degrades to below 90% at 30 °C after one quarter, reaching 80% at 35 °C, 60% at 40 °C, and 20% at 45 °C. Figure 4 shows the impact of temperature-affected oxytocin dose strength on the access ratio, confirming that the critical temperature is approximately 30 °C, after which the access ratio decreases dramatically through reduction in dose strength. Improper storage at higher temperatures (50 °C, 45 °C, 40 °C, 30 °C) leads to quicker degradation of dose strength [30, 31], requiring more oxytocin to achieve the same therapeutic effect. Therefore, the demand for oxytocin outweighs the supply in the latter weeks of a shipment cycle before the next shipment of the drug arrives (Fig. 4). The model’s results corroborate with the WHO’s survey on the stability of oxytocin, which illustrates a negligible degradation until approximately 30 °C, after which the rate of degradation increases significantly with increased temperature [33].

### Effect of literacy on decision to seek Care at a Facility

**Fig. 5:**
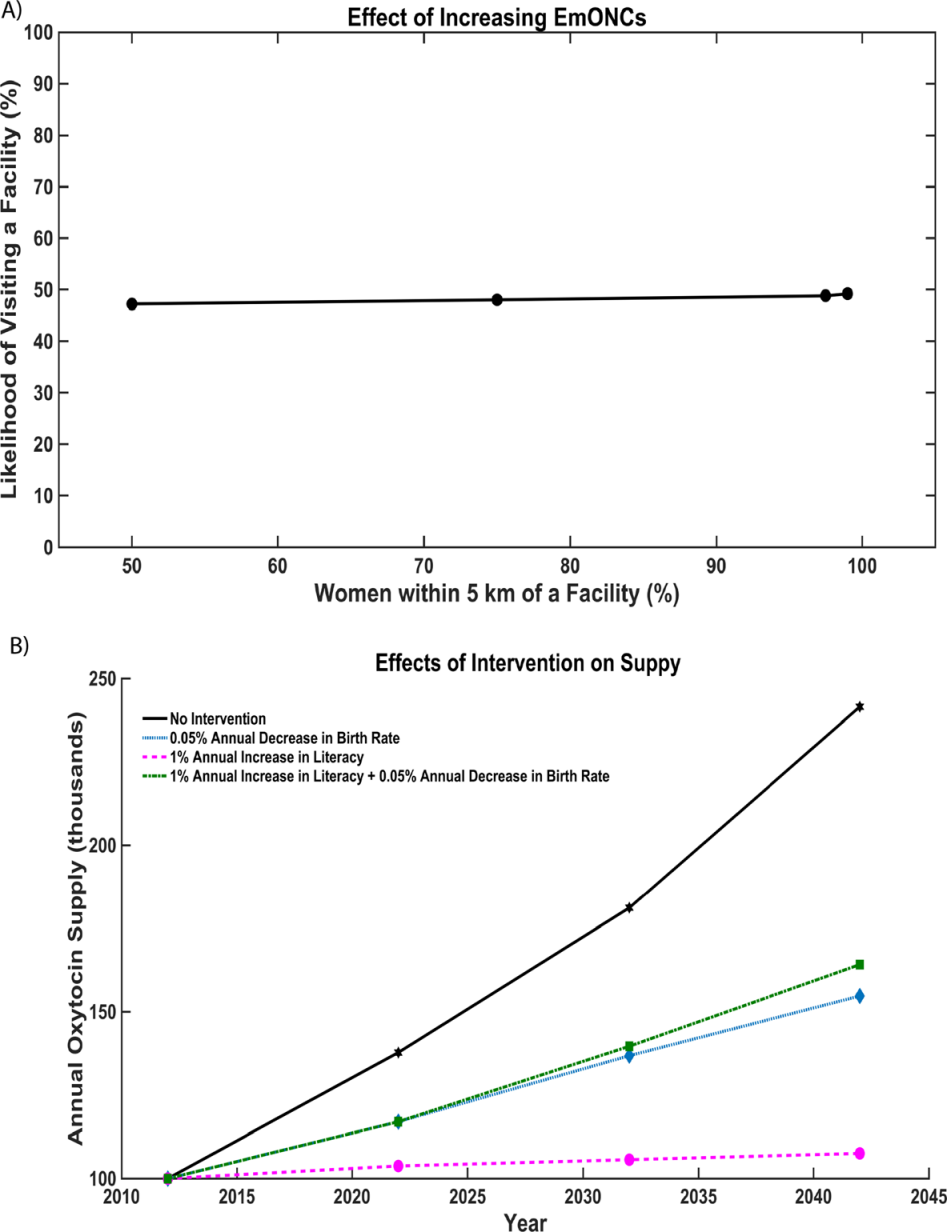
**a** Annual oxytocin supply needed to achieve access ratio of 1 under no intervention, decreasing annual birth rate by 0.5%, increasing literacy by 2% annually, both increasing literacy by 2% annually, and decreasing birth rate by 0.5% annually. **b** The effect of increasing the number of EmONCs on likelihood of visiting a faculty. Our model predicts that as the number of facilities are increased, the likelihood of visiting a faculty does not substantially increase. Even when the number of women who live within 5 km is doubled from 50% to nearly 100% the increase in likelihood remains flat

Literacy is one of the most successful predictors in determining whether women will deliver at a birthing facility. The predicted proportion of women visiting a health facility to deliver increased from 41% in the case of a completely illiterate population, to 53% in the case of a completely literate population. Zanzibar’s current literacy rate is 67% which corresponds to approximately 50% of women accessing a birthing facility [9]. Figure 5 demonstrates the effects of implementing initiatives to increase literacy, family planning, or both by tracking the annual supply required if literacy increased by 2% per year or the annual birth rate decreased by 0.5% per year or both literacy increased by 2% and birth rate decreased by 0.5%. The annual oxytocin supply to accommodate increased demand due to higher literacy is demonstrated in Fig. 5, with approximately 2000 more doses per year required by 2042 as compared to no intervention.

## Discussion

PPH is a major cause of maternal deaths. Oxytocin is recommended for use as a uterotonic to treat PPH, however access to oxytocin is impeded by both factors within the supply chain and expecting mothers’ socioeconomic characteristics. Our model combines these factors to analyze their effects on oxytocin access over time in Zanzibar.

A key observation we noted is that the number of drugs ordered by Zanzibar CMS is significantly higher than the amount needed to reach a high access ratio, based on our model’s calculations that account for natural degradation of oxytocin. Yet, stock-outs at public health facilities in Zanzibar, similar to facilities across sub-Saharan Africa, are common [30, 31]. This indicates that while the amount of doses ordered by CMS may be more than are required for Zanzibar, those doses are not all reaching patients in need. Doses that are lost or unaccounted for point to weaknesses in the health system that may be due to diversion, theft, or black market operations, which are consistent with reports from both the literature and the field [33].

Increased literacy was shown to slightly increase a woman’s likelihood to visit a health facility for delivery, thereby increasing the number of women seeking to deliver at health facilities - a safer route than home delivery [28]. Reducing distance was shown to have a negligible effect on choosing to access a facility for distances of 5 km or less, suggesting that building another EmONC to decrease the distance women will have to travel to a facility will likely not increase the proportion of women delivering at an EmONC. Furthermore, our model indicates that family planning interventions, combined with more stringent adherence to cold chain to protect against temperature-induced degradation, will help meet patient’s oxytocin needs at health facilities.

Our model also looks at access to quality oxytocin as a function of population growth. Zanzibar has a current population growth trend of 3% per year [12]. Accounting for current supply chain inefficiencies, our model predicts that by 2025, the access ratio will drop below 0.4 if the annual supply of oxytocin is not increased (Fig. 3). Therefore, to maintain an access ratio of 1.0, either the budget for oxytocin must be increased, or family planning interventions must be implemented to mitigate population growth (Fig. 3). Our model predicts that increased family planning interventions can decrease the oxytocin budget more than threefold in 30 years, with a 50% decrease in budget after just 10 years.

Model calculations for dose strength degradation are also consistent with the WHO data [15, 27]. The temperature-dependent oxytocin degradation rate would suggest that minor fluctuations in temperature, especially below 30 °C, do not significantly affect dose strength of oxytocin, and the viable oxytocin could still be used (Fig. 4). However, in the case of early degradation of oxytocin at high ambient temperatures, the access ratio plummets cyclically in the weeks before the shipment is renewed, resulting in severe deficits of oxytocin at health facilities a few weeks after a new shipment arrives.

### Assumptions used in developing the model

While the model is able to capture a number of essential features of the Zanzibari health system and provide new insights into access to quality oxytocin, it has a number of limitations, and conclusions drawn are subject to model validation. When assessing oxytocin quality, the model does not account for improper packaging or storage that would expose oxytocin to light and accelerate decay and assumes that there is no loss of drug quality during the manufacturing process. Furthermore, there is no standard to measure actual dose strength of oxytocin in hospitals, so administered dosage by health workers in practice may fluctuate per patient. The model assumes the therapeutic dose of oxytocin is 10 IU. Although studies have shown comparable clinical effectiveness between doses of 5 and 10 IU, the current model assumes loss of clinical effectiveness with loss of potency [34]. When assessing the socioeconomic determinants of women seeking to deliver at a health facility, the model uses a fixed hierarchy of wealth, literacy, and travel time, and does not account for any factor increasing or decreasing in predictive value over time. Further, we have not examined how cultural beliefs play a role in a woman’s decision to deliver at a health facility. The model only considers government funded supply of oxytocin, and excludes oxytocin supplied by private donors. We assume that oxytocin from the Central Medical Store is delivered only to the six EmONC facilities in Zanzibar based on demand from each facility, and these are the only facilities included in our model as they are the only ones required to have oxytocin at all times [35]. The model assumes an equal number of doses per shipment to the CMS or EmONC within a given year, as well as equal timing between shipments in that year, and does not examine the effects of transit time of oxytocin shipments between the CMS and EmONC [3]. Because of the lack of facility-level data, this model is currently most applicable to oxytocin access at a national level.

## Conclusions

Despite the limitations discussed, our model can predict access to oxytocin as a function of barriers within the supply chain as well as factors that impact patient access to birthing facilities. Providing a more comprehensive analysis of the strengths and shortcomings in the drug-to-patient path in Zanzibar, we hope that upon validation of this model policymakers will use it as a tool to improve the efficiency of their drug supply chain and the patient pathway to the facility, increasing availability of necessary maternal drugs for patients in need.

## Abbreviations

CMS: Zanzibar Central Medical Store
EmONC: Emergency Obstetric and Newborn Care
LMICs: Low and middle income countries
OR: Odds Ratio
PPH: Postpartum hemorrhage
TDHS: The Tanzania Demographic Health Survey
WHO: World Health Organization
